# Evaluation of a Locking Autocompression Screw Model in Pauwels Type-3 Femoral Neck Fracture: In Vitro Analysis

**DOI:** 10.3390/bioengineering9090464

**Published:** 2022-09-12

**Authors:** Vincenzo Giordano, Anderson Freitas, Robinson Esteves Pires, Leonardo Rigobello Battaglion, Mariana de Oliveira Lobo, William Dias Belangero

**Affiliations:** 1 Orthopedics and Traumatology Service Prof. Nova Monteiro, Hospital Municipal Miguel Couto, Rua Mario Ribeiro, 117, Rio de Janeiro 22430-160, RJ, Brazil; 2 Home Hospital Ortopédico e Medicina Especializada, SGAS Quadra 613-Conjunto C-Asa Sul, Brasília 70200-730, DF, Brazil; 3 Orthopedics and Traumatology Service, Hospital Regional do Gama, Área Especial No. 01, Brasília 72405-901, DF, Brazil; 4 Department of the Locomotive Apparatus, Universidade Federal de Minas Gerais (UFMG), Av. Pres. Antônio Carlos, 6627, Belo Horizonte 31270-901, MG, Brazil; 5 Ribeirão Preto School of Medicine, Universidade de São Paulo (FMRP-USP), Av. Dr. Arnaldo, 455, Ribeirão Preto 01246-903, SP, Brazil; 6 Department of Orthopedics and Traumatology, Faculty of Medical Sciences, Universidade Estadual de Campinas (UNICAMP), Rua Vital Brasil, 80, Campinas 13083-888, SP, Brazil

**Keywords:** femoral neck fracture, internal fixation, intramedullary fixation, finite element analysis

## Abstract

Femoral neck fractures in young adults are uncommon, resulting from high-energy trauma. Despite their infrequency in this population, there is higher rate of complications, especially in the more vertical fracture line, classified by Pauwels as a type-3 femoral neck fracture. The implant type is of paramount importance for maintaining anatomical reduction, since it must resist the deforming forces that act on the fracture. We comparatively evaluated two constructions of the novel locking autocompression implant (X-PIN and X-PIN+P) using the finite element method and previously established methods for treating Pauwels type-3 femoral neck fractures. Six fixation models were developed for the study: a dynamic hip screw (DHS), a DHS with an anti-rotation screw (DHS+P), the inverted triangle multiple cannulated screws construction (ASNIS), the multiple cannulated screws in an L-configuration (L), and the two models of the novel locking autocompression screw (X-PIN and X-PIN+P). Under the same conditions with a load of 2100 N, the following parameters were evaluated using SIMLAB^®^ software: the main maximum (Max P), main minimum (Min P), localized maximum P1 (Max P1), localized maximum P2 (Max P2), total displacement, localized displacement, rotation displacement, and von Mises stress. Compared to the DHS+P and ASNIS models, the X-PIN+P model presented, respectively, increases of 51.6% and 64.7% for Max P, 85% and 247% for Min P, and 18.9% and 166.7% for von Mises stress. Max P1 did not differ between the models, but Max P2 was 55% and 50% lower for X-PIN+P than ASNIS and L, respectively. All displacement values were lower for X-PIN+P than the other models. In this FEM testing, the X-PIN+P was superior to the other models, which was due to improvement in all parameters of stress distribution, displacement, and von Mises stress compared to models using a lateral plate (DHS and DHS+P) or not (ASNIS and L).

## 1. Introduction

Femoral neck fractures (FNF) in young adults are uncommon, accounting for 3% of all hip fractures, and usually result from high-energy trauma [1,2]. Treatment is focused on preserving the proximal extremity of the femur through anatomical reduction and stable internal fixation [3,4]. Despite their infrequency in the younger population, there is higher rate of femoral head osteonecrosis and nonunion, which directly contribute to a poor outcome and uneventfully are associated with reoperations and salvage procedures [2]. Although many factors have been shown to play a significant role in preventing these devastating complications, the quality of the reduction and its maintenance are the main recognized factors in reducing the risk of avascular necrosis of the head and nonunion of the femoral neck [2,4]. Epidemiological studies have shown up to a 59% nonunion incidence in FNF and from 12–86% avascular necrosis incidence in young patients after femoral neck fracture [2,5,6,7], with implant failure occurring in approximately 10% of cases in young patients [8].

The implant type is of paramount importance for maintaining anatomical reduction, since it must resist the deforming forces that act on the fracture focus [9]. The more unstable the fracture plane, the more critical this becomes, e.g., in Pauwels type-3 (P3) fractures, in which a dominant shear force is inherent to the fracture pattern, resulting in a higher rate of failure and nonunion [2,8,9,10]. The deformities often seen are varus angulation and inferior translation of the proximal femoral neck/head fragment, with failure often resulting after a non-anatomic reduction and inadequate fixation [2]. Thus, the search for effective methods of internal fixation has become the focus of scientific research over the years, resulting in the development of numerous implants that combine intra- and extramedullary characteristics [10,11,12,13,14].

Currently, the sliding hip screw, combined or not with an anti-rotation (or erotational) screw, is considered the standard implant in P3 FNF [7,10]. Several authors have shown that the sliding hip screw has less inferior femoral head displacement, less shearing displacement, and a greater load to failure when compared to multiple cannulated cancellous screws [9,10,11,12,13]. Bonnaire and Weber [15] observed that the sliding hip screw with the derotational screw presents the best mechanical environment for this challenging fracture pattern. Although the sliding hip screw has been found to be very effective in treating Pauwels type-3 femoral neck fractures, care should be taken in significantly comminuted fractures in a vertical orientation [16,17].

Despite the sliding hip screw’s superior mechanical strength to other extramedullary implants, problems related mainly to its inability to control rotation, especially when an additional derotational screw is not used, with varus subsidence and femoral neck shortening, which alter hip offset, have been reported in the literature [18]. This is mainly because the cephalic screw gradually slides, causing impaction of the fracture focus, which is greater in a malreduced fracture and in cases where the anti-rotation screw is not used. Thus, our hypothesis was that an implant which retained the main characteristics of existing systems (such as the cephalic screw and an intra- or extramedullary anchorage stop) but prevented the progressive collapse of the femoral neck during the healing process could minimize the rate of complications observed in young adult FNF. Indeed, some authors showed a reduced load-to-failure with the fixed-angle proximal femoral locking plate (PFLP), potentially minimizing femoral neck shortening and other complications [16,19,20]. Liporace et al. reported a nonunion rate of 8% for Pauwels type-3 FNF treated a PFLP, compared with 19%i n those treated with multiple cannulated screws [20].

The main objective of this study is to evaluate the biomechanical behavior of a locking autocompression screw, called X-PIN, and a variant (X-PIN+P) in P3 FNF using a finite element model (FEM). The secondary objective was to compare the results of this model with clinically established fixation methods for FNF.

## 2. Materials and Methods

**Figure 1:**
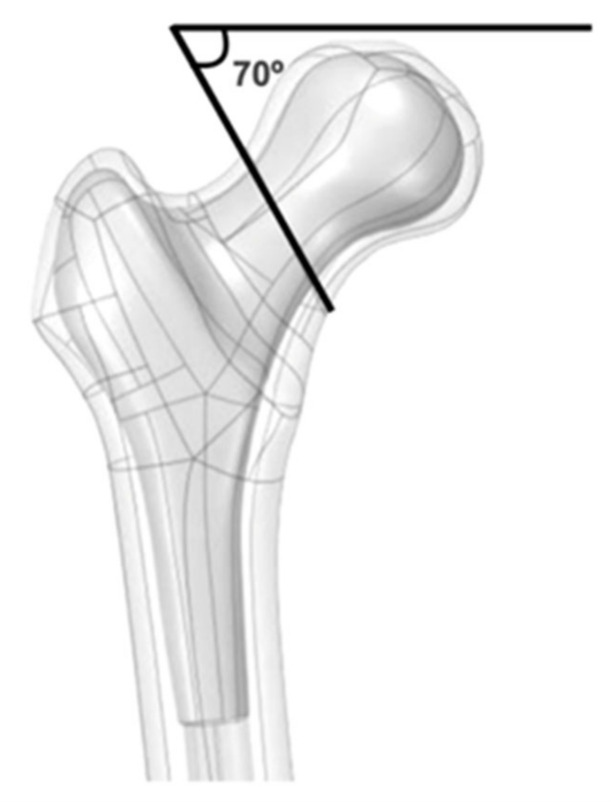
Representation of a Pauwels type-3 neck fracture. Note that the fracture line is oriented 70 degrees from the horizontal.

A fourth generation 3908 virtual model of the femur (Sawbones, Seattle, WA, USA) was used, which corresponds to a physical model with 17 pounds per cubic foot and characterizes the model as a young adult femur [21]. A full section was performed in the middle third of the femoral neck at an angle of 70° to the ground, which is considered a P3 fracture. Although some controversy still exists over the exact interpretation of Pauwels’ original description, current accepted interpretation is that type III fractures contain a fracture line oriented 70 degrees from the horizontal, which is considered inherently biomechanically unstable due to the shearing displacement [17]. No coefficient of friction value was added to the fracture surface (Figure 1).

**Figure 2:**
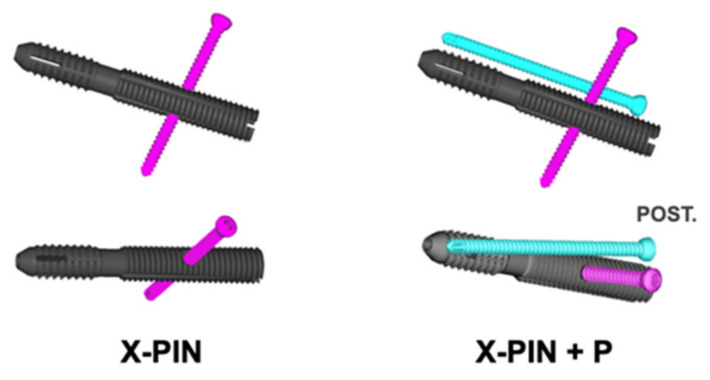
Representation of the X-PIN (left side) and the X-PIN+P. Observe that both extremities of the main screw have threads of different pitches and areas, allowing compression between the fractured fragments. A 4.7 mm locking screw (represented in pink) crosses the main screw from superior to inferior through a smooth hole and anchors bicortically. In the X-PIN+P model, a third screw (a fully threaded 4.7 mm screw, represented in blue) acts as a position screw from posterior to anterior along the femoral neck, transfixing the fracture without a sliding tunnel.

The X-PIN model features a main screw with 12.7 mm of distal diameter and 13 mm of proximal diameter, both extremities with threads of different pitches and areas, allowing compression between the fractured fragments, and a 4.7 mm locking screw that crosses the main screw through a smooth hole and anchors bicortically. The only change in the X-PIN+P model was a fully threaded 4.7 mm screw, positioned from posterior to anterior along the femoral neck, transfixing the fracture without a sliding tunnel, thus acting as a position screw (Figure 2).

For biomechanical comparison, we used a 135° dynamic hip screw (DHS) and a 7.0 mm cannulated screw, both manufactured by Hexagon Ltd. (São Paulo, SP, Brazil), in conformity with models approved for clinical use by the Brazilian National Regulatory Agency (ANVISA—Agência Nacional de Vigilância Sanitária).

**Figure 3:**
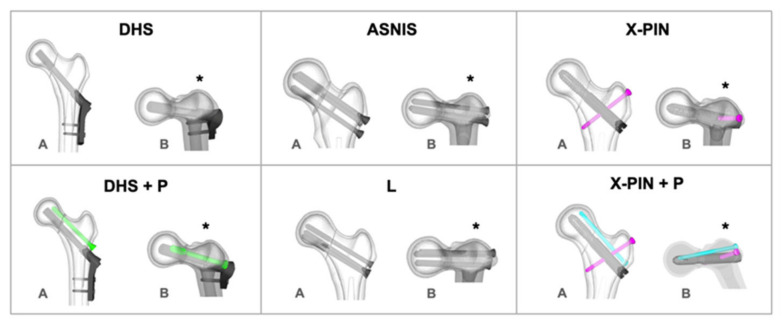
Illustrations of the included synthesis models. (*): posterior region; (**A**) coronal view of the proximal femur; (**B**) axial view of the proximal femur.

The models were abbreviated according to implant type: DHS, DHS with anti-rotation screw (DHS+P), inverted triangle multiple cannulated screws (ASNIS), multiple cannulated screws in an L-configuration (L), and X-PIN and X-PIN+P (the new autocompression system) (Figure 3).

For the simulations, the material properties, modulus of elasticity, and Poisson’s coefficient of each of the parts of the digital models (cortical bone, trabecular bone, and steel alloy) were previously defined. All metallic models in this study shared a common alloy (Table 1).

**Figure 4:**
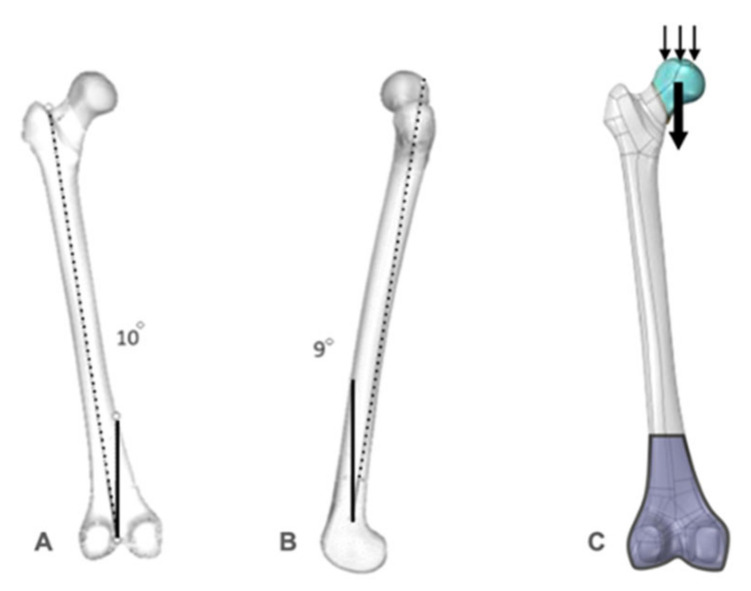
(**A**,**B**) Model of the virtual femur representing the inclination used in the tests; (**C**) the conditions and contours used during the test: load application area (blue with small arrows), load direction (larger arrow) and attachment area (purple).

After controlling the meshes of each part to certify perfect contact between the different structures, the regions of load application in the *X*, *Y*, and *Z* axes were selected. For the study, a 2100 N load was applied in the *Z* axis (which corresponds to the stress applied to the femur of a young adult weighing 70 kg in single-leg stance); no loads were applied to both the *X* and *Y* axes. Subsequently, the movement restriction regions (fixations) were delimited, marked in all directions of the *X*, *Y*, and *Z* axes to guarantee the stability of the system. A tetrahedral mesh formation was adopted for the meshes, and the models were tested with a 10° inclination in the *Z* axis (lateral) and a 9° inclination in the *Y* axis (posterior), with the load applied perpendicular to the ground in the superior region of the femoral head (Figure 4). The decision to adopt a single-leg stance was based on the observation that the bulk of the body weight mainly relies on the hip joint in this position, thus adequately representing the related forces acting around this joint when the whole body is standing on one foot [22].

Data on the displacement and stress in the FEM were collected, including the total principal maximum (Max P), localized maximum P1 (Max P1: area of greatest tension in the upper region of the femoral neck), localized maximum P2 (Max P2: area of greatest tension in the lateral peri-implant region of the femur), total principal minimum (Min P), presented as a negative value to represent the application axis, total displacement, localized displacement (displacement of the fracture focus), rotation deviation, and distribution of von Mises stress.

**Figure 5:**
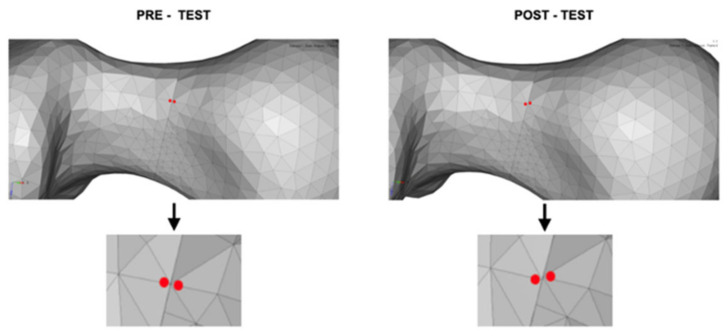
Upper left, nodes on the anterior face of the femoral neck selected for rotation deviation assessment (pre-test). Upper right, skewed nodes (post-test). In the two lower images, the pre- and post-test nodes at higher magnification. Observe that nodes are shown in red color in both figures.

The Max P1 and P2 values were calculated using the mean tension obtained in the nodes of the most affected region for each group. The rotation displacement was calculated by observing two coincident elements on opposite sides of the fracture before and after loading to evaluate their displacement (Figure 5).

The von Mises stress was captured in the synthesis material of the models and all the results are presented in absolute values and percentiles between the models.

## 3. Results

All results are presented for the DHS, DHS+P, ASNIS, L, X-PIN, and XPIN+P models in Table 2.

**Figure 6:**
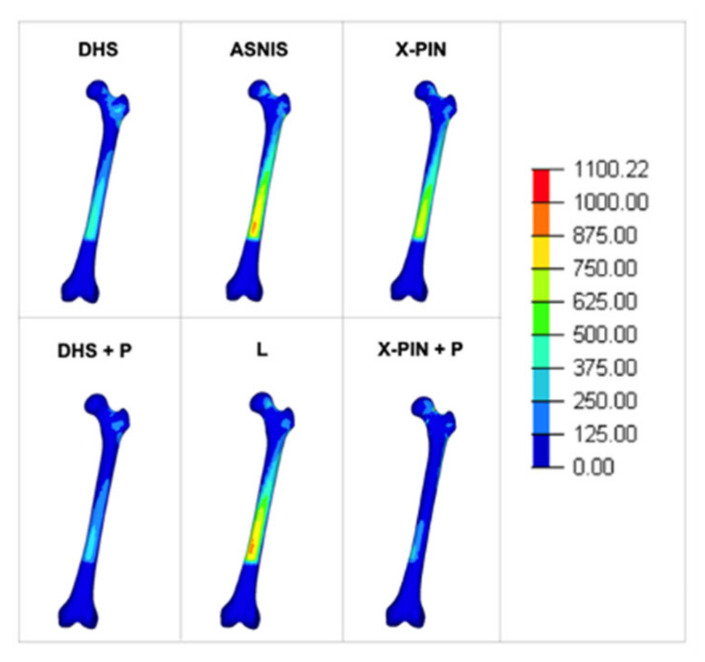
Maximum principal tension in the models.

The Max P values were 607, 711, 654.3, 501, 582, and 1078 MPa (Figure 6). The X-Pin+P model results were 51.6% and 64.7% higher than the DHS+P and ASNIS models, respectively.

**Figure 7:**
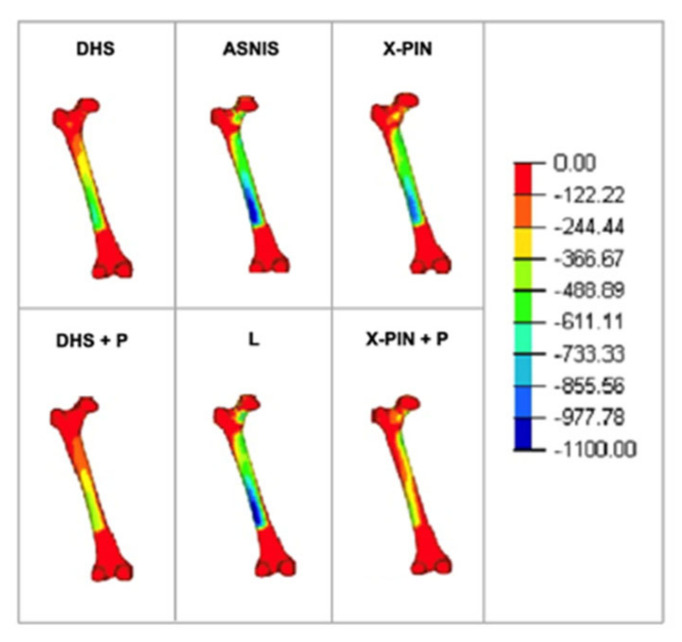
Minimum principal tension in the models.

The Min P values were “−765”, “−517”, “−276”, “−597”, “−856”, and “−959” MPa (Figure 7), with the X-PIN+P results 85% and 247% higher than DHS+P and ASNIS, respectively.

**Figure 8:**
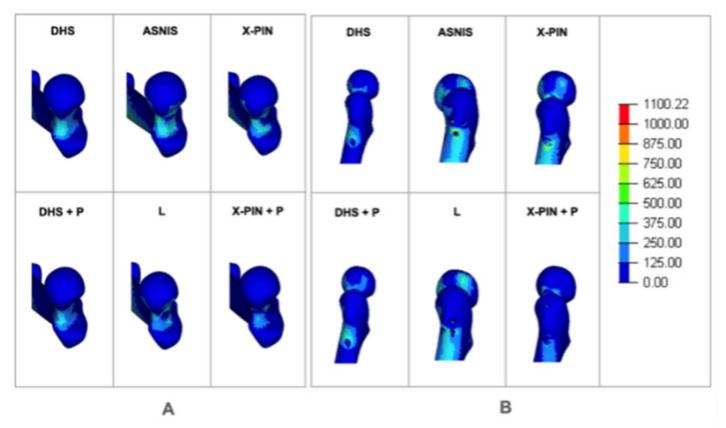
(**A**) Maximum local tension P1; (**B**) maximum local tension P2.

The Max P1 values were 29, 24, 23, 19, 28, and 25 MPa (Figure 8A). The models were balanced except for the L model, which had a lower value. The Max P2 values were 36, 27, 47, 42, 31, and 21 MPa (Figure 8B), with the X-PIN+P results 55% and 50% lower than ASNIS and L (the multiple cannulated screw models), respectively.

**Figure 9:**
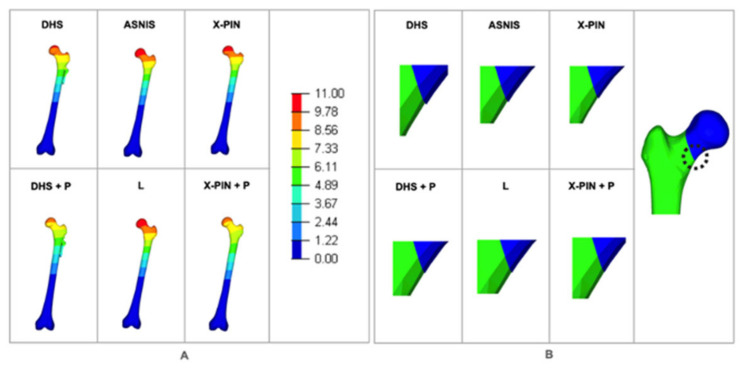
(**A**) Total displacement for each model; (**B**) localized displacement for each model.

The total displacement values were 8.4, 7.1, 9.2, 8.7, 9.0, and 2.4 mm. (Figure 9A), with the X-PIN+P results 66% and 73% lower than DHS+P and ASNIS, respectively. The localized displacement values were 2.1, 1.2, 2.0, 1.7, 2.6, and 0.8 mm (Figure 9B), while those of rotation deviation were 3.5, 1.5, 2.6, 2.3, 4.1, and 1.1 mm, with X-PIN+P lower than the other models.

**Figure 10:**
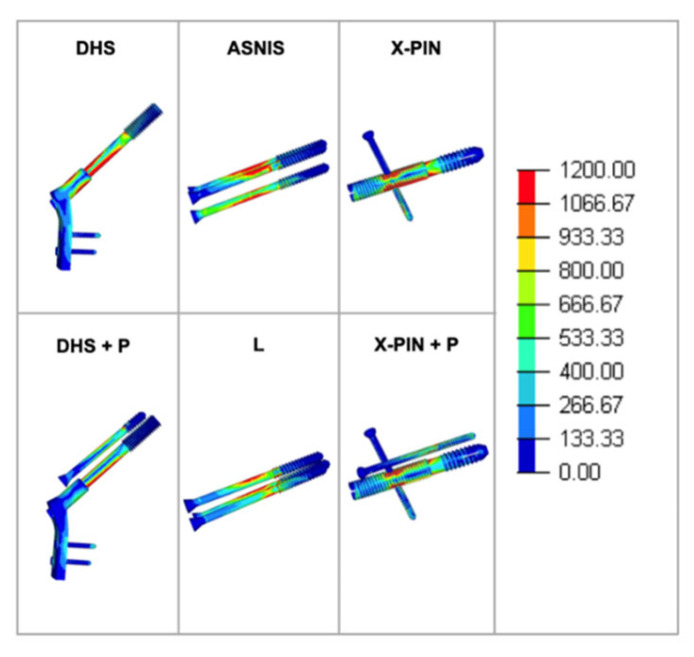
Von Mises stress in each group.

Finally, the von Mises values were 859, 1005, 448, 393, 955, and 1195 MPa, with the X-PIN+P results 18% and 166% higher than DHS+P and ASNIS, respectively (Figure 10).

## 4. Discussion

FNF in young adults have been the subject of several studies, especially P3 femoral neck fractures, due to the inherent instability of the vertical fracture line. Although the sliding hip screw is considered standard for this fracture pattern, the mechanical and biological failure rates are still not very acceptable. In addition, a large amount of bone is removed making later reconstructions difficult if required and there is a higher possibility of damaging the femoral head blood supply if the implant is imperfectly placed or there is a mechanical failure with varus collapse of the head [2]. Thus, it is necessary to study new designs and implant configurations for P3 FNF to mechanically improve the fixation, reducing or avoiding the deforming forces acting at the proximal extremity of the femur.

In this scenario, the X-PIN model was developed and tested in vitro using FEM, a fundamental tool for biomechanical investigations in orthopedics [23,24]. FEM verification focuses on the mathematical aspects, determining if the solution that has been computed is accurate, which has been validated in a number of studies for this purpose [14,21,25,26,27]. By allowing implant design projects and experimental tests, FEM provides understanding of the biomechanics of the bone synthesis material, effectively and efficiently evaluating several variables, such as implant variations and surgical techniques, to optimize not only the design, but also screening, prediction, and treatment in orthopedics [23]. In the present study, the new X-PIN device, especially when combined with an anti-rotation screw (X-PIN+P), was biomechanically superior to all other implants tested (DHS, DHS+P, ANIS, and L). In particular, the von Mises stress was higher and the total displacement was lower in the X-PIN+P group than in the other groups, which translates into greater stress absorption, especially when the values of these two measurements are observed together. The lower von Mises stress on the main screw of the X-PIN+P compared to the X-PIN was chiefly due to its function as a position screw, which acted to protect the main cephalic screw of the X-PIN. The position screw in X-PIN+P generally led to better stress distribution than the X-PIN in all analyses. In a certain way, this is observed with the sliding hip screw when used with an anti-rotation screw [15].

Recently, other systems in which the sliding screw is locked by another screw have been biomechanically and clinically investigated. Moon et al. [28] compared the stability of proximal fragment fixation and the mechanical characteristics in proximal femur models of a basicervical femoral neck fracture fixed by the new Femoral Neck System (FNS) vs. a sliding hip screw (the DHS). They used 20 composite femurs whose density was customized to young adult characteristics and a Pauwels type 2 femur neck fracture. No significant differences were found in the mean values of axial stiffness, rotation in the *X*, *Y*, and *Z* axes, cranial and axial migration of screws within the femoral head, or failure under vertical load. They concluded that a femoral neck system provides comparable biomechanical stability to the DHS for treating displaced femoral neck fractures in young adults. Unfortunately, their conclusion cannot be extrapolated to the more vertical P3 FNF. Davidson et al. [29] conducted a multicenter retrospective cohort analysis of patients treated with a femoral neck system, including 102 patients with a mean follow-up of 7 months. The fractures were classified according to the Garden system rather than Pauwels system. Overall, the revision rate was 9.2% (14 patients with implant cut-out, 10 with osteonecrosis of the femoral head, 8 with nonunion, and 8 requiring implant removal). The authors concluded that the femoral neck system is a safe treatment option for FNF, with failure rates comparable to those reported for other frequently used implants for this fracture type. Although we have not tested the femoral neck system, our findings cannot, even indirectly, be compared to those of these authors, since they did not use the Pauwels system.

Regarding distribution of the main maximum stresses in our study, the X-PIN+P was similar to the DHS and the DHS+P, but with better distribution. One hypothesis for this is the different cephalic screw locking system, with a screw inserted obliquely, more medially, and bicortically. Freitas et al. [30] found the same behavior in a similar study with a metaphyseal nailing system. Similarly, it can be interpreted that the oblique bicortical locking screw acts as a buttress mechanism, allowing angular stability and preventing proximal and distal migration of the cephalic screw.

It is interesting to note that the lack of a lateral plate on the femur (ASNIS and L) increased the load on this cortex, producing local weakness that is normally observed in clinical studies using only screws to fix P3 FNFs [31,32,33]. This could be a critical point in the new X-PIN system, since there is no lateral plate. However, the peri-implant stress in X-PIN and X-PIN+P was lower than that of the ASNIS and L models, showing that the lateral cortex of the femur is not weakened with the new system. Furthermore, the localized and rotation displacements were similar in the DHS+P and X-PIN+P groups, with lower values than the other groups. Finally, regarding distribution of the main maximum stresses, the X-PIN+P was similar to the DHS and the DHS+P, but with better distribution.

The main limitation of our study is the comparison between our findings with those of other biomechanical benchtop and clinical studies that did not use previous FEM analysis, which could be subject to several types of bias. The results of FEM can be influenced by several factors, such as the software, the types of meshes and elements, the minimum differences in conditions, and the contours. On the other hand, the results of benchtop tests have not always matched the clinical experience and a computer simulation might be indicated instead of idealized, simple models often used in experimental tests [34]. Augat et al. [34] stated that biomechanical studies typically have a higher sensitivity to detect a true difference between groups in a timely and cost-effective manner compared with clinical studies. Moreover, “if research precedes implant design, the results can lead to innovative solutions in a systematic, evidence-based strategy” [34]. Nevertheless, although our findings look promising for the X-PIN system, especially when combined with an anti-rotation screw (X-PIN+P), we strongly recommend extrinsic comparison to make sure that the biomechanical characteristics of these implants are reproducible under different conditions, such as benchtop tests or even clinical application in P3 FNF. In continued FEM analysis, we are formatting prototypes and conducting bench tests to be tested in other in vitro experiments under controlled conditions. Finally, as this is a controlled FEM analysis, we cannot assess the technical difficulty of placing the implant in a clinical situation. Future studies that will be carried out in phase 3 (clinical studies) will be able to show more assertively the step by step process of the X-PIN operative technique.

The main strength of our study is that stress distribution was better in X-PIN+P than DHS or DHS+P and, thus, was apparently superior to implants developed to treat the difficult condition of P3 FNF in young adults. Although we have not compared the X-PIN and X-PIN+P with the more contemporary implants such as the Synthes Femoral Neck System, the Rotationally Stable Screw-Anchor (RoSA) and the InterTan nail, biomechanical studies have shown that both mean axial stiffness and mean torsional stiffness of these implants were comparable to that of the DHS, especially when fractures were stabilized using the sliding hip screw system with a blade [35,36,37].

## 5. Conclusions

In FEM testing, the X-PIN+P was superior to the other models, which was due to improvement in all parameters of stress distribution, displacement, and von Mises stress compared to models using a lateral plate (DHS and DHS+P) or not (ASNIS and L).

## Figures and Tables

**Table 1:** Material properties.

Material	Material Properties	
	Modulus of Elasticity (MPa)	Poisson’s Coefficient (V)
Cortical bone	16,350	0.26
Trabecular bone	137	0.30
Syntheses (steel)	200,000	0.33

**Table 2:** Results for each model.

	Models					
Analysis	X-PIN	X-PIN+P	DHS	DHS+P	ASNIS	L
Max total (MPa)	582	1078	607	711	654.3	501
Min total (MPa)	−856	−959	−765	−517	−276	−597
Max P1 (MPa)	28	25	29	24	23	19
Max P2 (MPa)	31	21	36	27	47	42
Total displacement. (mm)	9	2.41	8.4	7.1	9.2	8.7
Local displacement (mm)	2.6	0.8	2.1	1.2	2	1.7
Rotational displacement (mm)	4.1	1.1	3.5	1.5	2.6	2.3
Von Mises stress (MPa)	988	1195	859	1005	448	393

Max P1: localized maximum P1 (area of greatest tension in the upper region of the femoral neck); Max P2: localized maximum P2 (area of greatest tension in the lateral peri-implant region of the femur).

## Data Availability

All relevant data are presented in the paper.
